# Process for Rapid Co-development of a Decision Aid Prototype for Population-wide Cancer Screening

**DOI:** 10.1177/0272989X251346894

**Published:** 2025-07-14

**Authors:** Odilon Quentin Assan, Claude Bernard Uwizeye, Hervé Tchala Vignon Zomahoun, Oscar Nduwimana, Wilhelm Dubuisson, Guillaume Sillon, Danielle Bergeron, Stéphane Groulx, Wilber Deck, Anik Giguère, France Légaré

**Affiliations:** Unité de soutien SSA Québec, Quebec, Canada; Chaire du Canada en décision partagée et mobilisation des connaissances, Quebec, Canada; VITAM – Centre de recherche en santé durable, Quebec, Canada; Unité de soutien SSA Québec, Quebec, Canada; Chaire du Canada en décision partagée et mobilisation des connaissances, Quebec, Canada; VITAM – Centre de recherche en santé durable, Quebec, Canada; Institut national d’excellence en santé et en services sociaux, Quebec, Canada; Unité de soutien SSA Québec, Quebec, Canada; Chaire du Canada en décision partagée et mobilisation des connaissances, Quebec, Canada; VITAM – Centre de recherche en santé durable, Quebec, Canada; Ministère de la Santé et de Services sociaux du Québec, Quebec, Canada; Ministère de la Santé et de Services sociaux du Québec, Quebec, Canada; Ministère de la Santé et de Services sociaux du Québec, Quebec, Canada; Ministère de la Santé et de Services sociaux du Québec, Quebec, Canada; Centre intégré de santé et de services sociaux de la Gaspésie, Quebec, Canada; VITAM – Centre de recherche en santé durable, Quebec, Canada; Unité de soutien SSA Québec, Quebec, Canada; Chaire du Canada en décision partagée et mobilisation des connaissances, Quebec, Canada; VITAM – Centre de recherche en santé durable, Quebec, Canada

**Keywords:** decision aid, rapid, culturally adapted, co-development, process, Quebec, shared decision making

## Abstract

Decision aids (DA) are more likely to be adopted if co-developed with stakeholders and culturally adapted. Using the DEVELOPTOOLS Reporting Checklist, we describe a process for rapid co-development of a culturally adapted DA prototype for population-wide cancer-screening programs. Our systematic, collaborative, and iterative methodology had 7 phases: 1) set up the process by adopting best governance practices (e.g., identify and engage stakeholders, adapt our collaborative DA design process, validate development process), with governance comprising 20 individuals from a wide range of sectors including at least 2 citizens; 2) identify and analyze existing DAs relevant to the cancerscreening of interest by conducting a systematic review; 3) share results with stakeholders and make recommendations; 4) formulate Quebec-specific DA content and consult stakeholders including users by conducting e-Delphi surveys; 5) co-design a prototype with stakeholders, including users, following international DA standards; 6) translate the DA using translation–back translation approaches and deploy; and 7) knowledge mobilization (KMb) using end-of-grant and integrated KMb activities. Using the User-Centred Design 11-Item Measure (UCD-11), our proposed process scored 10 of 11 on the UCD-11. Overall, we expect this new co-developed process to ensure that good-quality, user-centered, and culturally adapted DAs for cancer screening are produced within reasonable timeframes. We also expect it to foster the adoption of the DAs.

**Highlights:**

- We report on a 7-step process for collaborating with various stakeholders to create a culturally adapted decision aid (DA) prototype for deciding about cancer screening in Quebec, Canada.
- The process includes:
  ○ Making sure the DA prototype design includes users and other interested parties and reflects their needs, perceptions, values, and preferences.
  ○ Finding and analyzing existing DAs on cancer screening to decide what ours should include
  ○ Respecting international standards and criteria for DA design
  ○ Repeated rounds of expert consensus about the exact content, with revisions between each round
- This method could help the rapid creation of DAs shaped by users’ interests and will ultimately encourage shared decision making.

## Background

Patient decision aids (DAs) serve to engage individuals in shared decision making by providing them with key knowledge about their health condition, presenting options for a health-related decision based on best evidence, and helping them identify what matters to them.^1–4^ As of October 2024, the Ottawa Hospital’s Decision Aid Library Inventory (DALI) listed more than 817 published and unpublished DAs, an increase of 672 since 2010, suggesting an exponential increase of their use.^5^ The use of DAs has been significantly associated with better patient–clinician communication, increased knowledge, alignment between informed values and care choices, decreased decisional conflict, fewer undecided people, and less decision regret.^6^ However, there are still challenges to ensuring DA adoption by health systems.^7–9^ One potential avenue for increasing their uptake is to better align their development process with the values and needs of stakeholders, including patients, citizens, and health care professionals.^9,10^ Early models of DA development did not emphasize user engagement in the development process,^11,12^ but more recent studies have shown that it is a key factor in DA adoption and use.^13,14^

A recent systematic review presented a portrait of existing DA development practices (including which users are involved and at which steps)^15^ using the user-centered design (UCD) framework.^16^ According to this framework, a user-centered DA development process may involve 3 main steps: 1) understanding users’ needs, 2) developing or refining a DA prototype, and 3) observing prospective users’ interactions with the DA. The review recommended an 11-item tool (UCD-11)^10^ in addition to the 20-item Reporting Checklist (DEVELOPTOOLS) to assess the user-centeredness of the DA development process.^17^ Briefly, the UCD-11 items are 1) pre-prototype involvement: whether potential end users were involved in any steps that help developers understand these end users and their needs in developing the prototype, evaluating prototypes or final versions or using the DA, or whether they were asked their opinion of the DA at all; 2) iterative responsiveness: whether the development process had 3 or more iterative cycles and whether changes between cycles were explicitly reported; and 3) other expert involvement: whether health professionals were consulted in any way, before the first prototype, between initial and final prototypes, or were involved in expert panels. Other authors have pointed out that DAs should be culturally adapted to encourage their adoption,^18^ outlining procedures for their cultural adaptation and validation. However, achieving user and stakeholder involvement in DA development processes according to UCD-11 principles may seem complex and challenging. In one study, only 35% of 325 DA studies reported patient involvement in at least 1 step of the development/refining of the prototype.^15^ Another assessment of 283 DA development processes found they earned a mean score of 6.45 out of the 11 UCD principles.^17^ A flexible process that can be adapted to a variety of situations has been recommended.^17^

Practically, DA developers should decide on their own process, taking into account the issues of need, time, and cost. The development process of DAs is typically time-consuming, and there have been calls for generalized insights or templates that would allow for speedier and more efficient production.^19^ The Ottawa Decision Support Framework^20^ is one such template that contributed to fulfilling this need.^21^ The urgency of the COVID pandemic highlighted the need for speedier development of DAs.^21^ In fact, experienced developers can develop good-quality, time-critical DAs using abbreviated processes. For example, during COVID-19, experienced developers were able to develop 2 DAs in 2 wk.^19,21^

Cancer is the leading cause of death in the province of Quebec, ahead of cardiovascular diseases, and Quebec is projected to have the highest cancer rate in Canada in 2024, with an estimated 558 cases per 100,000 people.^22^ There is a strong call for increased prevention, including cancer screening by the public health system.^23^ Clinical practice guidelines increasingly recommend that the decision to undergo cancer screening should be made using a shared decision-making approach.^24^ In the context of cancer screening, patients face several choices, such as choosing among the available screening tests^6,25–27^ or deciding whether or not to undergo any of them. They have to weigh up the benefits of cancer screening (increased chance of survival) with its possible harms (false-positive test results, overdiagnosis).^28^ Shared decision making has been shown to increase individual satisfaction with cancer screening,^6,24,29,30^ and good-quality DAs can help achieve this.^6,24^

Both the high prevalence of cancer and the urgency of the COVID pandemic highlight the need for speedier development of DAs.^21,31^

In this report, we therefore describe the process we designed with users for rapid co-development of culturally adapted DA prototype for a population-wide cancer screening program. We also provide a quality assessment on how our approach complies with UCD-11 principles and the additional items from the DEVELOPTOOLS Reporting Checklist.

## Co-development Process

### Context

**Figure 1:**
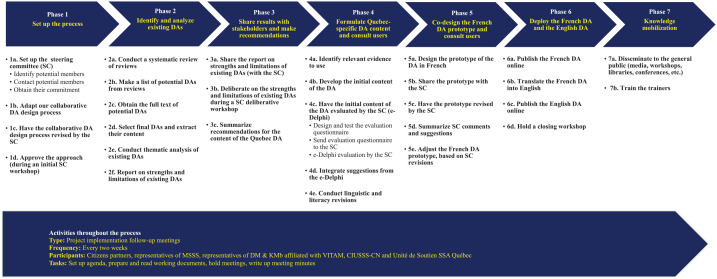
Phase-by-Phase and Step-by-Step Summary of the Process for Rapid Co-development of a Culturally Adapted Decision Aid Prototype for Population-Wide Cancer-screening Programs. CIUSSS-CN, Centre Intégré Universitaire de Santé et de Services Sociaux de la Capitale Nationale; DA, Decision Aid; DM & KMb, Canada Research Chair in Shared Decision Making and Knowledge Mobilisation; MSSS, Ministère de la Santé et des Services sociaux (Quebec Ministry of Health and Social services); SC, Steering Committee; Unité de Soutien SSA Québec, Unité de Soutien au système de santé apprenant Québec; VITAM, VITAM – Centre de recherche en santé durable.

Canada has 14 health care systems. Each province and territory manages health care for its own residents, and the federal government provides services for specific populations such as military personnel and certain Indigenous Canadians. Quebec is the second largest province and health care system (9M inhabitants)^32^ with the largest French-speaking population (84%).^33^ Trends show increasing cancer incidence in Quebec.^34^ Quebec’s Ministry of Health and Social Services (MSSS) aims to ensure the continuous improvement of the quality of screening services including effective decision support based on best practices.^34^ In 2019, the MSSS expressed the desire to foster shared decision making by contributing to the development of DAs. To develop the prototype of a DA for a specific cancer-screening program, we codesigned a multiphase, collaborative, and iterative process (Figure 1, Appendix 1). We situate this process in step 2 of the UCD framework: developing/refining the DA prototype.^16^ It mobilizes the knowledge and experience of various Quebec stakeholders at all steps from beginning to end. We adopted the Canadian Institute of Health Research (CIHR)’s Integrated Knowledge Mobilization (iKMb) approach^35^ as well as the Strategy for Patient-Oriented Research (SPOR) principles^36^ to engage stakeholders. We report our co-development process according to the DEVELOPTOOLS Reporting Checklist.^17^

### Process

Briefly, this process for the co-development of population-wide screening decision aids has 7 phases with multiple iterations. It is designed to engage stakeholders and to yield culturally adapted decision aids. The process includes several iterations and lasts approximately 6 mo. The process involves a steering committee of 20 stakeholders, including users, with a wide range of expertise and backgrounds. This committee guides all phases of the process but meets formally twice. Day-to-day follow-up is delegated to a multidisciplinary operations subcommittee of the steering committee.

### Phase 1: Set up the Process

The purpose of this phase was to set up a steering committee to direct the project and to seek the perspectives of a variety of stakeholders involved in cancer screening. Members were identified collaboratively based on a snowball sampling approach, enabling us to reach specific groups rapidly and efficiently.^37,38^ In addition, the inclusion and consultation of users in our steering committee enabled us to avoid lengthy user field testing.^24^ Based on their networks, the MSSS and a team from the Canada Research Chair on Shared Decision Making and Knowledge Mobilization (DM & KMb) identified prime potential members. Selected prime members were then asked to recommend other potential members. We aimed to enroll about 20 Steering Committee (SC) members, including citizens (2 or more), prevention and screening program managers and policy makers (6 or more), and health professionals, for example, cancer experts, primary and specialized health care professionals (6 or more), researchers in shared decision making and DA development (4 or more), epidemiologists (4 or more), information specialists (1 or more), and experts in knowledge mobilization (4 or more). Invited citizens were patients who had experienced cancer, family members (1 or more), friends, caregivers (1 or more), and surrogates or citizens who had experienced the cancer-screening process (1 or more). Targeted operating ecosystems included the community, public institutions, universities, hospitals, professional associations, and research centers. Identified members were contacted for their consent to participate. All members were sent a confidentiality and consent form to engage voluntarily in the process. A first workshop was organized to share and adopt the project objectives, methodology, and administration as well as to clarify roles and responsibilities and funding sources. Prior to this workshop, we shared our collaborative DA design process with all SC members and revised it according to their suggestions. The role of the SC was to validate, guide, and monitor the DA co-development process through all phases. It operated through periodic meetings (including deliberative workshops) and document reviews (including a review of our collaborative DA design process, DA content, DA prototype, and project reports). However, to ensure day-to-day follow-up, an Operations Subcommittee of the SC met on a biweekly basis to discuss any problems with project implementation and potential solutions, for a total of 12 formal meetings. It included citizens partners, representatives from the MSSS, and representatives from the Canada Research Chair on Shared Decision Making and Knowledge Mobilization (DM & KMb).

### Phase 2: Identify and Analyze Existing DAs

**Figure 2:**
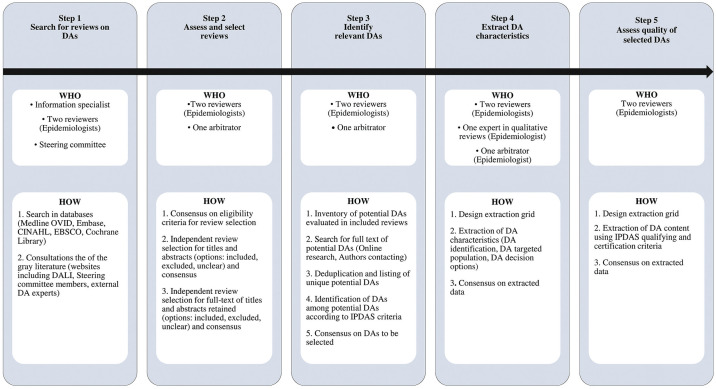
Step-by-step Summary of Phase 2—Systematic Review of Reviews to Identify and Analyze Existing DAs. DA, decision aid; DALI, Decision Aid Library Inventory; IPDAS, International Patient Decision Aid Standards.

The purpose of this phase was to make an inventory of existing DAs worldwide on the screening decision in question and analyze their strengths and limitations. Thanks to the International Patient Decision Aid Standards (IPDAS) minimum standards of DA qualifying criteria (6 items) and certification criteria (10 items), we were able to rapidly assess their quality to ensure that our DA replicated their strengths and avoided their weaknesses.^39^ To identify existing DAs, we conducted a systematic review of reviews (SRR) using the Cochrane Collaboration methodology for guidance^40^ (Figure 2). We report this SRR according to the Preferred Reporting Items for Systematic Reviews and Meta-Analysis (PRISMA).^41^

#### Search for existing DAs: systematic review of reviews

We set eligibility criteria following the PICOS approach; Population (P), Intervention (I), Comparator (C), Outcome (O), Study design (S).^42^ We first searched in MEDLINE (OVID), EMBASE, CINAHL (EBSCO), and Cochrane Library bibliographic databases from their inception. An information specialist performed a search strategy using MEDLINE and revised it with an expert in systematic reviews. The revised search strategy was then translated into the other bibliographic databases. More details on the bibliographic database search including search strategy can be found in Appendix 2. Second, we searched the gray literature. We consulted Web sites of specialized institutions such as the Ottawa Patient Decision Aid Library Inventory (DALI) (www.decisionaid.ohri.ca). We also asked the SC members and international collaborators about any DAs they may be aware of. Endnote was used to import all citations and eliminate duplicates following the method proposed by Bramer.^43^ Single citations were then imported into an Excel file for the process of selection.

#### Select reviews and evaluate their full texts

Two reviewers conducted a 3-step process to select relevant reviews using an Excel file: 1) consensus on eligibility criteria, 2) independent selection of titles and abstracts based on the eligibility criteria, 3) selection of relevant full text of reviews from selected or unclear titles and abstracts. Discrepancies were discussed at each step by the reviewers until a consensus was reached. Otherwise, the final decision fell to the arbitrator.

#### Identify DAs

Potential DAs were first identified independently from selected reviews. The same potential DA could be reported in more than 1 review. Duplicates were then eliminated. At this stage, we referred to “potential” DAs (we were not yet sure if every tool labeled a DA qualified as a DA according to IPDAS criteria). Then we searched for the full text or video of the potential DAs, either by searching online or by contacting the authors. Full texts or videos of potential DAs were reviewed by both reviewers to eliminate those that did not meet IPDAS criteria. DAs in languages other than French and English were excluded but archived.

#### Extract and analyze data on characteristics of identified DAs

Two reviewers independently extracted data from identified DAs using a piloted Excel grid. They extracted general characteristics (title of the DA, year of publication, names of authors, institution and country of development, date of last update, format of the DA, language of publication); characteristics of the populations targeted by the DA (average age, gender, targeted sociocultural group); and targeted health condition and decision options. DAs available in a PDF or Web-based format were directly extracted, while video-based DAs were first transcribed by a qualitative research expert prior to extraction. For all characteristics extracted, discrepancies were discussed between the 2 reviewers until a consensus was reached. Otherwise, the final decision fell to an arbitrator. Data on general characteristics were summarized using descriptive analysis. Frequencies and percentages were used for the categorical variables and means, standard deviations, medians, interquartile ranges, and ranges for the continuous variables. DAs were the units of analysis. SAS software (SAS Institute Inc.) was used for analysis.

#### Assess quality of potential DAs

Two reviewers assessed the quality of identified DAs according to the IPDAS criteria.^39^ They used a piloted Excel grid to standardize the assessment. This assessment was based on the 6 qualification and 10 certification IPDAS criteria.^39^ Qualification criteria served to confirm if a potential DA was really a DA, whereas certification criteria certified that the DA presented no risk of harmful bias in decision making. At this stage, we first extracted the crude content of the DA as it responded to each specified IPDAS criterion, rating it with “Yes,” “No,” or “Unclear” depending on how the extracted content fulfilled the IPDAS criterion. The more “Yes” answers there were, the more likely the DA was qualified and/or certified as a DA. After a conclusive pilot test, the 2 reviewers proceeded to data extraction independently. Subsequently, they discussed discrepancies until a consensus was reached; otherwise, the final decision fell to an arbitrator.

#### Conduct thematic analysis of identified DAs

Two reviewers independently codified the extracted content using an inductive analysis method. This allowed us to generate themes and categories of the extracted crude content of the DA. For example, the criterion “the patient decision aid describes the health condition or problem” was broken down into further subthemes such as anatomy and physiology of the organ involved in the health condition, the natural history of the cancer targeted by the DA, its epidemiology, and its risk factors. This categorization helped to better structure the specific content of the DA under development. Subsequently, discrepancies during codification were discussed between the 2 reviewers until a consensus was reached.

#### Report on the strengths and limitations of identified DAs

We drew up a report on the characteristics and the strengths and limitations of selected relevant DAs. The report also included suggestions for thematic content for the Quebec DA according to IPDAS criteria and the thematic analysis described above. The summary of this report was discussed at the SC deliberative workshop.

### Phase 3: Share Results with Stakeholders and Make Recommendations

The purpose of this phase was to inform SC members of project progress and to discuss the strengths and limitations of the DAs identified in phase 2. We opted for a deliberative workshop^44^ as it could 1) be organized within a reasonable time frame, 2) enhance SC members’ knowledge about DA content that meets IPDAS criteria and how DA developers operationalize them worldwide for the targeted cancer, and 3) reinforce their engagement in the process.^44^ We conducted a 1-h session based on a guide for deliberative dialogues.^44^ We then provided SC members with a summary of the results of phase 2 and invited them to participate in the deliberative workshop. During the workshop, we invited them to share their experiential knowledge on relevant Quebec-specific contextual, cultural, or formatting elements to include in a Quebec DA about cancer screening and make recommendations. A facilitator with experience of working with different stakeholders, including citizens, health professionals, researchers, and policy makers organized discussions. We provided information that was balanced and easy to understand, and there was equal opportunity for everyone to share their views. We also invited participants to continue to share their views even beyond the workshop. After the workshop, we drafted a report on the ideas and recommendations that emerged for the Quebec DA content and sent it to SC members for revision and approval.

### Phase 4: Formulate Quebec-Specific DA Content and Consult Users

In this phase, our purpose was to produce a first draft of the French DA content for use in Quebec. We based this draft on the thematic analysis of existing DAs (phase 2), on recommendations by the SC (phase 3), on a rapid review of the evidence, and on information relevant to cancer screening for the Quebec population as prioritized by SC members through committee consultations and the e-Delphi process described below.

#### Prioritize relevant evidence-based information

A critical issue in this phase was the identification of all relevant evidence-based information to be presented in the DA. To quickly obtain a synthesis of recent evidence, we conducted a rapid review according to Cochrane guidance.^45,46^ In descending order, we prioritized systematic reviews with meta-analyses, randomized trials, or large population cohort studies.^46^ We looked for information on screening outcomes such as screening test performance, decrease in incidence and mortality following screening, and other advantages and disadvantages of each decision option. We co-conducted with an information specialist a systematic search of the scientific literature based on criteria developed using the PICOS approach.^42^ In descending order, evidence was preferably from Quebec, elsewhere in Canada, or from contexts that were as close as possible to the Quebec screening context. A summary of retained references was submitted to SC members for consensus on which data to present and how to present it in the DA.

#### Develop the initial content of the DA

We formerly designed a DA format structured according to the IPDAS criteria.^39^ Two reviewers used this format to develop the content of the present DA. For each IPDAS criterion, we developed content according to the thematic analysis (phase 2) and aligned it with the SC recommendations (phase 3) and further relevant evidence-based information derived from the e-Delphi described below.

#### Evaluate the initial content of the DA through an e-Delphi process

The 2 reviewers who developed the initial content of the DA conducted a Delphi process with SC members to evaluate it. No outside consulting took place. We opted for an e-Delphi (electronic) to reach them remotely throughout Quebec and to gather their opinions systematically while avoiding desirability bias as much as possible.^47^ We enlisted SC members as panelists for the Delphi as they represented a wide variety of expertise and experience regarding screening for the targeted cancer in Quebec.

We adapted our method from the RAND/UCLA Appropriateness Method.^48^ We invited panelists to participate through individual e-mails to maintain the highest degree of anonymity. We did not predefine a participation rate. A first reviewer used the Google Forms platform to develop the e-Delphi questionnaire that included all 48 sections of the DA, and a second checked it. Each section was assessed with a 5-point Likert scale: *totally disagree* (1), *somewhat disagree* (2), *neither disagree nor agree* (3), *somewhat agree* (4), or *totally agree* (5). They tested the questionnaire for functionality and adjusted it before sending to panelists. Only the initial DA content was provided as background material. For each section, panelists could make comments or rephrase the item whenever their evaluation was *totally disagree*, *somewhat disagree*, or *neither disagree nor agree*. The questionnaire also included questions on sociodemographic characteristics (expertise, age, gender). A reminder was sent midway to the deadline to ensure maximum participation.

We anticipated 1 round for the e-Delphi given that all SC members had contributed their recommendations during the deliberative workshop in phase 3. We followed up with individual respondents if needed, for example, on questions of literacy, reformulation, format, or reconsideration of the evidence-based information used. We stopped the process if we 1) reached 55% of participants giving a rating of 3 (*neither agree nor disagree*) or more for each item^49^ or if the MSSS judged that the iteration process was exhausted. When no further suggestions were received from SC members, we assembled all opinions from their comments and suggestions.

We then summarized responses from the e-Delphi together with sociodemographic characteristics. We used thematic analysis for commentaries and suggestions and descriptive analysis for sociodemographic characteristics (percentage, mean, standard deviation for age, percentage for expertise and gender) and item scores.

#### Integrate suggestions from the e-Delphi

The first reviewer integrated the comments and suggestions to obtain a revised draft. Then the second reviewer validated the revision to ensure all comments and suggestions were considered. Because comments and suggestions could steer the revision in different directions, the 2 reviewers used a consensus process to reach agreement in case of discrepancy. Although there are no standards for the length of a DA, we thought it preferable to have a concise, short DA with literacy level accessible to the population while aligning with IPDAS criteria.^50^ We discussed the final product with MSSS communication experts to check for grammar, vocabulary, literacy, and length. We gave feedback to panelists through a thank-you e-mail that indicated the participation rate and next steps and shared the adjusted content of the DA.

### Phase 5: Co-design the French DA prototype and Consult Users

The purpose here was to use the post-Delphi French DA content to co-design a French DA prototype that complied with MSSS communication rules while taking into account the IPDAS criteria for a balanced presentation of information.^50^ The graphic designers from MSSS designed a prototype of the DA and shared it with the Operations Subcommittee of the SC for initial review and improvement. The Operations Subcommittee of the SC then returned it to the graphic designers if improvements were required. Once requested improvements were made, the Operations Subcommittee of the SC shared the prototype with the whole SC. SC members reviewed the content and format of the DA prototype. We summarized their suggestions and communicated it to the graphic designers for integration in the prototype. We anticipated at least 4 iterative cycles of revision. We followed up with the designers through online meetings or e-mail.

### Phase 6: Deploy the French DA and the English DA

The purpose of this phase was to refine and translate the French DA into English and to disseminate both versions.

#### Diffusion of the French DA

At this step, we published the French DA on the MSSS Web site.^51,52^ As well, members of the SC received the link to the DA and were invited to share it with their communities.

#### Translate the French DA into English

We conducted the English translation according to the Transcultural Adaptation Process Guidelines.^53^ This 5-step process (Appendix 3) covered the entire content of the DA. Feedback from users of the French DA, as well as from the translators and users of the English DA, were collected by the MSSS, analyzed, and integrated into each version as necessary by the Operations Subcommittee of the SC. Once done, the English DA was also published on the MSSS Web site^54,55^ and shared with the SC. SC members were then invited to share it with their communities.

#### Hold a closing workshop

Once the 2 versions of the DA were published, the Operating Subcommittee organized a closing workshop for all SC members. Its purpose was to review the phases completed, summarize levers and challenges encountered, and suggest solutions to informing the co-development of future DAs, with particular emphasis on user involvement.

### Phase 7: Knowledge Mobilization

The purpose of this phase was to conduct several knowledge translation activities with the new DA. First, it was integrated into government screening pathways.^56^ The MSSS disseminated the DA by informing its contacts in the network of managers and health care professionals of regions and health care establishments where cancer screening occurs. The MSSS also integrated it into training modules on best screening practices designed for health care professionals^57^ as well as in video vignettes on screening that target the general public.^58,59^ The DA was also diffused on the Learning health support unit Quebec Web site^60–63^ and through social media^64^ and was registered on the DALI inventory in English^65,66^ and in French.^67,68^ It was also disseminated through research center newsletters as well as at scientific conferences.^69,70^ Further dissemination took place through train-the-trainer courses and activities for the public. We also plan to disseminate it through our workshops on shared decision making in the Quebec library network, all co-developed and facilitated with and by citizen partners.^71^

## Assessment of User Centeredness

Table 1 summarizes the correspondence between our process and the DEVELOPTOOLS Reporting Checklist^17^ as well as its scores on the UCD-11 items.^10^ Our process achieved an overall score of 10 out of 11 on UCD-11 (2/2 for pre-prototype involvement, 4/5 for iterative responsiveness, and 4/4 for other expert involvement). This score was higher than the mean self-reported score of 54 DA developers reviewed in 2021 (mean = 9.62 [*s* = 1.16])^17^ and complied with 19 of the 20 DEVELOPTOOLS Reporting Checklist items.^17^ Table 2 also highlights the similarities between our process and the user-centered processes defined in a systematic review of 283 DA development processes.^17^ Most items met the medium or maximum engagement standards; that is, all phases involved significant end-user participation.

**Table 1:** Correspondence between Our Process Phases, the DEVELOPTOOLS Reporting Checklist, and Its Scores according to UCD-11 Factors and Corresponding Items

UCD-11 Items and DEVELOPTOOLS Checklist Items	Yes/No	Our Process Phase	UCD-11 Measure Scoring: Yes = 1, No = 0	Reporting Checklist Additional Question(s)	Reported?
UCD-11 items (1 to 11)					
Factor: Pre-prototype involvement			2 out of 2		
1. Were potential users (patients, caregivers, family and friends, surrogates) involved in any steps to help understand users (e.g., who they are, in what context might they use the tool) and their needs?	Yes	All phases	1	If yes, what did you do (e.g., interviews, focus groups, surveys)? How many users of each type were involved in each of these steps?	Yes
2. Were potential users (patients, caregivers, family and friends, surrogates) involved in any steps of designing, developing, and/or refining a prototype?	Yes	Phases 3, 4, 5	1	If yes, what did you do (e.g., co-design workshops)? How many users of each type were involved in each of these steps?	Yes
Factor: Iterative responsiveness			4 out of 5		
3. Were potential users (patients, caregivers, family and friends, surrogates) involved in any steps intended to evaluate prototypes of the tool or a final version of the tool?	Yes	Phase 5, 6	1	If yes, what did you do? How many users of each type were involved in each of these steps?	Yes
4. Were potential users (patients, caregivers, family and friends, surrogates) asked their opinions of prototypes of the tool or a final version of the tool in any way?	Yes	Phases 3, 4, 5, 6	1	If yes, what did you do? How many users of each type were involved?	Yes
5. Were potential users (patients, caregivers, family and friends, surrogates) observed using the tool in any way?	No	N/A	0	If yes, what did you do? How many users of each type were involved?	No
6. Did the development process have 3 or more iterative cycles?	Yes	Phases 4, 5, 6	1	If yes or no, how many cycles did you have?	Yes
7. Were changes between iterative cycles explicitly reported in any way?	Yes	Phases 4, 5, 6	1	If yes, what did you do?	Yes
Factor: Other expert involvement			4 out of 4		
8. Were health professionals asked their opinion of the tool at any point?	Yes	All phases	1	If yes, what did you do?	Yes
9. Were health professionals consulted before a first prototype was developed?	Yes	Phases 2, 3, 4	1	If yes, what did you do?	Yes
10. Were health professionals consulted between initial and final prototypes?	Yes	Phases 5, 6	1	If yes, what did you do?	Yes
11. Was an expert panel involved?	Yes	All phases	1	If yes, who was involved?	Yes
Additional elements in DEVELOPTOOLS Reporting Checklist (12 to 20)					
12. Was a formal advisory panel of users involved?	Yes	All phases	N/A	If yes, what kind of panel was it and how was the panel assembled and involved?	Yes
13. Were users (patients, caregivers, family and friends, surrogates), health professionals, and other relevant stakeholders involved as members of the research team?	Yes	All phases	N/A	If yes, who were the users, health professionals, and other relevant stakeholders? What perspectives did they bring, and how were they involved?	Yes
14. Were members of populations marginalized by social norms and policies involved?	Yes	All phases	N/A	If yes, what populations were involved, how were they recruited, and how were they involved?	Yes
15. How many users (patients, caregivers, family and friends, surrogates) and health professionals were involved in total and of each type?	Yes	Phase 1	N/A	How many people of each type were involved?	Yes
16. Does the tool have a defined purpose?	Yes	N/A	N/A	What is the purpose of the tool?	Yes
17. Is the tool intended to be used in a particular context?	Yes		N/A	In what context is the tool intended to be used?	Yes
18. Were any methods used to facilitate sharing of perspectives between groups?	Yes	Phases 3, 4	N/A	If yes, what methods were used?	Yes
19. Were users (patients, caregivers, family and friends, surrogates) involved from the outset of the project?	Yes	Phase 1	N/A	At what point were users involved?	Yes
20. Were translation and cultural adaptation used to render the patient decision aid available to users across languages and cultures?	Yes	Phase 6	N/A	If yes, what was done?	Yes

N/A, not applicable; UCD-11, User-Centred Design 11-Item Measure.

**Table 2:** Similarities (highlighted in blue) between Our Decision Aid Design Process and Minimal, Medium, and Maximal User-Centeredness in DA Development Processes^a^

Item	Minimal Process^b^ (>70% of 283 Projects, First Quartile of Counts)	Medium Process^a^ (40%–69% of 283 Projects, Median of Counts)	Maximal Process^a^ (5%–39% of 283 Projects, Third Quartile of Counts)
Iterative nature of overall design and development process	• Process is iterative, with at least 2 cycles^c^ • Changes between cycles are not explicitly noted or reported	• Process is iterative, with at least 3 cycles • Changes between cycles are not explicitly noted or reported	• Process is iterative, with at least 4 cycles • Changes between cycles are explicitly noted and reported
Development steps for understanding users (patients, family members, caregivers, surrogates) and their contexts	• Conduct literature review	• Conduct literature review • Conduct informal needs assessment^d^ with 30 users	• Conduct literature review • Conduct informal needs assessment with 43 users^e^ • Conduct formal needs assessment with 44 users • Observe existing processes (e.g., ethnography) with 56 users
Development steps for developing and refining prototype patient decision aid	• Develop prototype	• Conduct content and format review prior to prototyping with 14 users • Develop prototype	• Develop and/or validate underlying model • Storyboard or wireframe design • Adapt or translate content and format for different cultural groups with 38 users • Conduct content and format review prior to prototyping with 25 users • Develop prototype
Development steps for observing users’ (patients, family members, caregivers, surrogates) interactions with the prototype	• Conduct pilot or usability testing with 15 users	• Review content and format of developed prototype with 20 users • Conduct pilot or usability testing with 28 users	• Review content and format of developed prototype with 30 users • Conduct pilot or usability testing with 45 users • Conduct additional rounds of pilot or usability testing with 40 users
People involved as users (patients, family members, caregivers, surrogates)	• People currently facing this decision	• People currently facing this decision	• People currently facing this decision • People who faced this decision in the past • People who may face the decision in the future • People who are members of populations marginalized by social norms and policies are specifically involved
What is evaluated?	• Efficacy	• Efficacy • One or more of feasibility, acceptability, satisfaction, and usability	• Efficacy • One or more of feasibility, acceptability, satisfaction, and usability • Implementation
How is evaluation done?	• Users are asked their thoughts and opinions of the patient decision aid • Impact of patient decision aid is assessed (e.g., through knowledge questionnaires)	• Users are asked their thoughts and opinions of the patient decision aid • Impact of patient decision aid is assessed (e.g., through knowledge questionnaires) • Users are observed interacting with the patient decision aid	• Users are asked their thoughts and opinions of the patient decision aid • Impact of patient decision aid is assessed (e.g., through knowledge questionnaires) • Users are observed interacting with the patient decision aid
Sociodemographic data reported about users(patients, family	• Age • Sex and/or gender • Education	• Age • Sex and/or gender • Education	• Age • Sex and/or gender • Education
members, caregivers, surrogates) involved	• Clinical Characteristics	• Clinical characteristics • Race and/or ethnicity	• Clinical characteristics • Race and/or ethnicity • Literacy or health literacy
Involvement of health professionals who are not members of the research team	• 5 health professionals who are not members of the research team are involved in some way	• 13 health professionals who are not members of the research team are involved in some way • Health professionals are asked their thoughts and opinions of the patient decision aid	• 26 health professionals who are not members of the research team are involved in some way • Health professionals are asked their thoughts and opinions of the patient decision aid • Impact of patient decision aid on clinical practice is assessed (e.g., on shared decision-making practices) • Health professionals are observed interacting with the patient decision aid
Advisors	• None	• Expert panel (panel of academics, health professionals, etc.)	• Expert panel (panel of academics, health professionals, etc.) • Formal links with a specific patient or consumer organization • Users are involved in an advisory capacity (as individual advisors, as part of an advisory panel, as members of the research team)

DA, decision aid.

a Adapted from Witteman et al.^17^

b We consider these processes applicable to the design and development of new patient decision aids without an existing template and in the absence of urgent needs. For a design and development process to meet the standards of any of the 3 processes, it should typically include all steps in that column, acknowledging that, sometimes, certain steps may not be included for valid reasons (e.g., health professionals may not be involved if the patient decision aid is explicitly intended to be used only by the patient; projects that use in-depth qualitative methodologies may have fewer numbers of people involved.^17^

c An iterative cycle is defined as, “Your team developed something and showed it to at least 1 person outside the team before making changes in response to their reactions or feedback. Each new cycle leads to a version of the tool that has been revised in some small or large way.”^17^ In our process, the initial content of the DA developed by 2 reviewers is submitted to validation by the subcommittee (iteration 1). Then, the revised version is submitted to the steering committee through the e-Delphi process (iteration 2). Personalized follow-ups are then undertaken with specific respondents (1 to 4 rounds of follow-up, iteration 3). Iterations 2 and 3 lead to the final content that is used to develop the DA prototype. Other iterations based on the prototype are made to obtain the French DA.

d Here, users are defined as patients, family members, caregivers, or surrogates. While we acknowledge that health professionals may be deeply involved in the use of patient decision aids, ultimately, patient decision aids are designed for the people whose health or family may be affected by the decision. Therefore, for brevity, we refer to *users* when referencing patients, family members, caregivers, or surrogates and *health professionals* when referencing the people who provide health care to users.^17^

e A formal needs assessment was defined as per the authors’ reports, meaning that authors reported conducting a “needs assessment.” An informal needs assessment was defined as using the methods of needs assessments (e.g., interviewing patients to explore what support they would like to have when making the decision) without naming it as such.^17^

## Discussion

We described a process for rapid co-development of a culturally adapted DA prototype for a population-wide cancer-screening program. This process has 7 phases with at least 4 iteration cycles involving a multidisciplinary stakeholders’ committee that includes users. For each phase, we have described in detail the purpose, the stakeholders, and the methodology used. Using this process, we have already co-developed DAs on lung cancer screening and on colorectal cancer screening, which are available on the Web site of the Quebec Ministry of Health and Social Services and are currently being used by the population. Both have acceptable IPDAS scores, according to the DALI inventory,^65,66^ of the Quebec Ministry of Health and Social Services and the development process scores high on the UCD-11. The implementation of this process has led to the following reflections.

First, from the stakeholder’s perspective, the process was deemed acceptable and feasible for implementation and adaptation. It enables continuous consensus building and agreement among various stakeholders at every phase. It also ensures constant involvement, participation, and follow-up by both users and policy makers.^72^ The process could be adapted for co-developing DAs for other kinds of screening, such as cardiovascular disease screening, and prenatal screening, as well as for other care contexts in which shared decision making is applicable such as vaccination, treatments, surgery, diagnostic exploration, or clinical trials.^5,73^ The process could also be used to update or adapt existing DAs. Some phases could be shortened. For example, phase 2—identify and analyze existing DAs—could focus on the most recently published or more local DAs. Phase 3—share results with stakeholders and making recommendations—could also be shortened, particularly if there was already a consensus on the initial DA content. However, this might extend the time required to reach a consensus on the content to be evaluated by e-Delphi in phase 4—formulate Quebec-specific DA content and consult users. In addition, phase 4 could be shortened by focusing the rapid review of the literature solely on the years following the initial co-development. In the context of an emergency, experienced DA developers could identify existing DAs and present them to the steering committee at phase 3 of the deliberative workshop. A simple Delphi, e-mail exchanges, or individual consultations could also replace the e-Delphi at phase 4—formulate Quebec-specific DA content and consult users. However, this approach might diminish the richness of the feedback.

Second, the classic snowball sampling technique used in phase 1 to set up the steering committee offered significant advantages in terms of time saving. The use of an extensive network of personal contacts accelerated the recruitment process, enabling the formation of a sufficiently large and diverse participant group to accurately represent the targeted cancer-screening ecosystem in Quebec.^37,38^ Phase 2 of the process, the review and thematic analysis of existing DAs, achieved both speed and efficacy in fulfilling IPDAS criteria and providing content at the same time. Thematic analysis of the content of existing DAs provided insight into how DA developers worldwide have populated the IPDAS criteria in their own settings. In the context of cancer screening, DA developers generally include the anatomical–histological definition of cancer, its natural history, the signs and symptoms of cancer, its risk factors, and its epidemiology in the “definition of the health problem” criterion, giving us a clear idea of our potential content. Thus, this phase ensured that the initial draft of the DA both conformed to IPDAS standards and already contained relevant evidence on the health topic addressed. Our proposed phase 2 thus simultaneously increases the standards and quality of the content of the initial DA. Phase 3—share results with stakeholders and make recommendations—also saved time. In the short term, it rapidly bridged the gap between evidence and practice, facilitating the mutual exchange of opinions and enabling a consensus on appropriate content for a Quebec DA.^44^ In addition, the choice of a rapid review to obtain the evidence-based information saved time. Other authors reported they developed 5 decision box prototypes in 5 mo using rapid reviews.^46^ The e-Delphi technique used to evaluate the initial content of the DA is also known for being time saving and expediting consensus building.^74,75^ This phase was also accelerated by the preexistence of a group of experts, namely, the steering committee set up during phase 1.

Third, the process provides a model for user-centered development of DAs,^13^ that is, early involvement of stakeholders, consideration of their views on different versions of the DA, and their feedback on changes made to all intermediate versions.^15^ It also demonstrates the co-development of DAs adapted to a specific cultural context as recommended in recent studies on adapting DAs.^18,76,77^ Procedures included gathering users and other stakeholders from the diverse health care settings where population-based cancer screening is offered in Quebec into a steering committee. Their feedback as well as our rigorous translation procedure (Appendix 3) ensured that the content reflected the specific cultural idioms of our context (for example, in Quebec, there are specific screening pathways and culturally specific ways of referring to every aspect of screening, in both English and French). A rapid review found that co-production of DA content and processes was a key facilitator of DA implementation.^9^ The same review also noted that cultural adaptation led to better fit with the local setting and addressed the needs of more end users. Authors also noted that collaborative development with stakeholders who may be affected by the adoption of the DA contributes to its successful integration into routine practice and screening pathways.

Our development process has several strengths. First, to our knowledge, this is the first description of a process for the rapid co-development of a DA prototype for population-based cancer screening for use in Quebec. Second, each phase is guided by approaches, frameworks, methodologies, methods, guidelines, or international criteria that reflect best practices while taking into account time constraints and the need for rapidity. For example, we adopted the IPDAS criteria for improving the balance of the information (pros and cons) in our DA. This approach ensures we obtained a DA that meets the highest international standards.^50^ We also systematically reviewed and assessed the strengths and weaknesses of existing DAs, according to IPDAS criteria, to build on existing DAs and save time. Third, a multidisciplinary steering committee including users and other stakeholders was mandated to guide the entire process. Fourth, overall, the process requires the ability to continuously reconcile the opinions of all these stakeholders throughout multiple iterations. This guidance by multidisciplinary stakeholders and ongoing consensus among them about how to proceed ensured user-centeredness and cultural adaptation. It also created a shared sense of ownership and endorsement as well as legitimizing content and ultimately fostering DA adoption. For example, deliberative dialogues are known to build individual and community capacity over the medium term, promoting adoption and change, engaging public decision makers and officials in meaningful action. This strategy is recommended when implementing or scaling innovations in health and social services.^78^ Fifth, 2 pillars underpin shared decision making: evidence-based information and users’ values and preferences. Our process addresses both. We propose a systematic literature search of the evidence, but we also consider the experiential knowledge of users and other SC members. We also consult external experts when necessary. Sixth, the process mobilizes different approaches to enriching the DA content and prototype with the different opinions of stakeholders. Finally, along with other frameworks, such as the Patient Decision Aids Implementation Toolkit,^79^ our process could be adapted for co-developing DAs for other specific chronic disease screening such as cardiovascular disease screening and prenatal screening as well as for other care contexts in which shared decision making is applicable such as vaccination, treatments, surgery, diagnostic exploration, or clinical trials.^5,73^ The process could also be used to update or adapt existing DAs.

Our development process has some limitations. All co-production of DAs should follow a robust process, but this can be highly time- and resource-consuming, especially a DA that is adapted to a specific cultural and linguistic context or is designed to be scaled in the future. Ours was no exception. For example, it required human resources for managing the collaboration and building consensus throughout the process, as described. However, we attempted to identify areas where time and resources could be reduced in future iterations. In addition, the process will soon be more streamlined through a digital platform that has been developed under the leadership of a member of our steering committee.^80^ This platform will allow us to automate some of the phases of our process, particularly in terms of consensus building. It will provide a transparent and convenient forum for direct and real-time interaction between all stakeholders. Second, although stakeholders and citizens have benefited from our DAs through our knowledge mobilization activities, we feel that there is more to be done to increase awareness of the DAs and to improve shared decision making for cancer screening.

## Conclusion

This process offers a step-by-step guide to co-developing DAs for cancer screening ready to be implemented in routine practice. In a context in which cancer is the leading cause of death, this process ensures that evidence-based and culturally adapted DAs for screening can be rapidly and rigorously developed in partnership with users for immediate deployment and future scaling. With the growing interest in DA development, it would be useful to design a standard process that can continually incorporate the latest guidelines for use in various contexts. A robust, adaptable, and continually updated standard process will ensure that future DAs are aligned with current best practice. In turn, this may improve the effectiveness of DAs, leading to more informed decisions and better outcomes for patients. Our process serves as a baseline for the development of such a standard process.

## Supplemental Material

sj-pdf-1-mdm-10.1177_0272989X251346894 – Supplemental material for Process for Rapid Co-development of a Decision Aid Prototype for Population-wide Cancer Screening Supplemental material, sj-pdf-1-mdm-10.1177_0272989X251346894 for Process for Rapid Co-development of a Decision Aid Prototype for Population-wide Cancer Screening by Odilon Quentin Assan, Claude Bernard Uwizeye, Hervé Tchala Vignon Zomahoun, Oscar Nduwimana, Wilhelm Dubuisson, Guillaume Sillon, Danielle Bergeron, Stéphane Groulx, Wilber Deck, Anik Giguère and France Légaré in Medical Decision Making
