# Supplementary material for: Process for Rapid Co-development of a Decision Aid Prototype for Population-wide Cancer Screening
Source: Med Decis Making. 2025 Jul 14;45(7):775–93. doi: 10.1177/0272989X251346894 (PMC12413505; doi:10.1177/0272989X251346894)
Supplement: sj-pdf-1-mdm-10.1177_0272989X251346894 – Supplemental material for Process for Rapid Co-development of a Decision Aid Prototype for Population-wide Cancer Screening [file sj-pdf-1-mdm-10.1177_0272989X251346894.pdf]

UNITÉ DE SOUTIEN SSA | QUÉBEC
Ensemble pour un système de santé qui apprend

VITAM
CENTRE DE RECHERCHE EN SANTÉ DURABLE

Chaire de recherche du Canada sur la décision partagée et la mobilisation des connaissances

UNIVERSITÉ LAVAL

Process for rapid co-development of a decision aid prototype for population-wide cancer screening

Odilon Quentin Assan, Claude Bernard Uwizeye, Hervé Tchala Vignon Zomahoun,
Oscar Nduwimana, Wilhelm Dubuisson, Guillaume Sillon, Danielle Bergeron,
Stéphane Groulx, Wilber Deck, Anik Giguère, France Légaré

May 2025

Purpose of this document

Summarize the process for rapid co-development of a decision aid prototype for population-wide cancer screening

Process for rapid co-development (Summary)

Phase 1. Set up the process

Phase 2. Identify and analyze existing DAs

Phase 3. Share results with stakeholders and make recommendations

Phase 4. Formulate Quebec-specific DA content and consult users

Phase 5. Co-design the French DA prototype and consult users

Phase 6. Deploy the French DA and the English DA

Phase 7. Knowledge mobilization

Activities throughout the process. Project implementation follow-up meetings

DA: Decision aid

Process for rapid co-development (Phase 1)

Phase 1	Phase 2	Phase 3	Phase 4	Phase 5	Phase 6	Phase 7
Set up the process	Identify and analyze existing DAs	Share results with stakeholders and make recommendations	Formulate Quebec-specific DA content and consult users	Co-design the French DA prototype and consult users	Deploy the French DA and the English DA	Knowledge mobilization

- Set up a multidisciplinary steering Committee
- Adapt our collaborative DA design process
- Approve the approach (during an initial steering committee workshop)

Process for rapid co-development (Phase 1)

Phase 1 – Set up the process

Phase 2 – Identify and analyze existing DAs

Phase 3 – Share results with stakeholders and make recommendations

Phase 4 – Formulate Quebec-specific DA content and consult users

Phase 5 – Co-design the French DA prototype and consult users

Phase 6 – Deploy the French DA and the English DA

Phase 7 – Knowledge mobilization

Set up a multidisciplinary steering Committee

Adapt our collaborative DA design process

Approve the approach (during an initial steering committee workshop)

SC members (about 20)

- Researchers in shared decision making and DA development (4 or more)
- Health professionals (cancer experts, primary and specialized healthcare professionals) (6 or more)
- Epidemiologists (4 or more)
- Prevention and screening program managers and policy makers (6 or more)
- Information specialist (1 or more)
- **Citizens (2 or more)**
- Experts in knowledge mobilization (4 or more)

→ **Steering Committee (SC)**

Process for rapid co-development (Phase 1)

Phase 1	Phase 2	Phase 3	Phase 4	Phase 5	Phase 6	Phase 7
Set up the process	Identify and analyze existing DAs	Share results with stakeholders and make recommendations	Formulate Quebec-specific DA content and consult users	Co-design the French DA prototype and consult users	Deploy the French DA and the English DA	Knowledge mobilization

Set up a multidisciplinary steering Committee

Adapt our collaborative DA design process

Approve the approach (during an initial steering committee workshop)

SC members (about 20)

- Researchers in shared decision making and DA development (4 or more)
- Health professionals (cancer experts, primary and specialized healthcare professionals) (6 or more)
- Epidemiologists (4 or more)
- Prevention and screening program managers and policy makers (6 or more)
- Information specialist (1 or more)
- **Citizens (2 or more)**
- Experts in knowledge mobilization (4 or more)

→ **Steering Committee (SC)**

Targeted operating ecosystems
- Community
- Public institutions
- Universities
- Hospitals
- Professional associations
- Research centres

Goal of targeting diverse operating ecosystems
To be as representative as possible.

Role of the steering committee
Validate, guide, and monitor the process.

Operating mode of the steering committee
- Periodic meetings
- Document review

UNITÉ DE SOUTIEN SSA | QUÉBEC
Ensemble pour un système de santé qui apprend

VITAM
CENTRE DE RECHERCHE EN SANTÉ DURABLE

Chaire de recherche du Canada sur la décision partagée et la mobilisation des connaissances

UNIVERSITÉ LAVAL

Process for rapid co-development (Phase 2)

Phase 1 – Set up the process

Phase 2 – Identify and analyze existing DAs

Phase 3 – Share results with stakeholders and make recommendations

Phase 4 – Formulate Quebec-specific DA content and consult users

Phase 5 – Co-design the French DA prototype and consult users

Phase 6 – Deploy the French DA and the English DA

Phase 7 – Knowledge mobilization

Phase 2 steps:

1. Conduct a systematic review of reviews on existing DAs
2. Conduct thematic analysis of existing DA
3. Report on strengths and limitations of existing DAs

Process for rapid co-development (Phase 3)

Phase 1	Phase 2	Phase 3	Phase 4	Phase 5	Phase 6	Phase 7
Set up the process	Identify and analyze existing DAs	Share results with stakeholders and make recommendations	Formulate Quebec-specific DA content and consult users	Co-design the French DA prototype and consult users	Deploy the French DA and the English DA	Knowledge mobilization

Phase 3:

1. Share the report on strengths and limitations of existing DAs
2. Deliberate on the strengths and limitations of existing DA during a steering committee deliberative workshop
3. Summarize recommendations for the content of the Quebec DA

Process for rapid co-development (Phase 4)

Phase 1 – Set up the process

Phase 2 – Identify and analyze existing DAs

Phase 3 – Share results with stakeholders and make recommendations

Phase 4 – Formulate Quebec-specific DA content and consult users

- Identify relevant evidence to use
- Develop the initial content of the French DA
- Have the initial content of the French DA evaluated by the SC
- Integrate suggestions from the e-Delphi
- Conduct linguistic and literacy revisions

Phase 5 – Co-design the French DA prototype and consult users

Phase 6 – Deploy the French DA and the English DA

Phase 7 – Knowledge mobilization

Process for rapid co-development (Phase 5)

Phase 1	Phase 2	Phase 3	Phase 4	Phase 5	Phase 6	Phase 7
Set up the process	Identify and analyze existing DAs	Share results with stakeholders and make recommendations	Formulate Quebec-specific DA content and consult users	Co-design the French DA prototype and consult users	Deploy the French DA and the English DA	Knowledge mobilization

Phase 5 steps:

1. Design the prototype of the DA in French
2. Share the prototype with the SC
3. Summarize SC comments and suggestions
4. Adjust the French DA prototype, based on SC revisions

Process for rapid co-development (Phase 6)

- **Phase 1** – Set up the process
- **Phase 2** – Identify and analyze existing DAs
- **Phase 3** – Share results with stakeholders and make recommendations
- **Phase 4** – Formulate Quebec-specific DA content and consult users
- **Phase 5** – Co-design the French DA prototype and consult users
- **Phase 6** – Deploy the French DA and the English DA
  - Publish the French DA online
  - Translate the French DA into English
  - Publish the English DA online
  - Hold a closing workshop
- **Phase 7** – Knowledge mobilization

UNITÉ DE SOUTIEN SSA | QUÉBEC
Ensemble pour un système de santé qui apprend

VITAM
CENTRE DE RECHERCHE EN SANTÉ DURABLE

Chaire de recherche du Canada sur la décision partagée et la mobilisation des connaissances

UNIVERSITÉ LAVAL

Process for rapid co-development (Phase 7)

Phase 1	Phase 2	Phase 3	Phase 4	Phase 5	Phase 6	Phase 7
Set up the process	Identify and analyze existing DAs	Share results with stakeholders and make recommendations	Formulate Quebec-specific DA content and consult users	Co-design the French DA prototype and consult users	Deploy the French DA and the English DA	Knowledge mobilization

- Disseminate to the general public (media, workshops, libraries, conferences, etc.)
- Disseminate to healthcare professionals through training modules on good screening practices
- Train the trainers

Process for rapid co-development (Activities throughout the process)

Phase 1	Phase 2	Phase 3	Phase 4	Phase 5	Phase 6	Phase 7
Set up the process	Identify and analyze existing DAs	Share results with stakeholders and make recommendations	Formulate Quebec-specific DA content and consult users	Co-design the French DA prototype and consult users	Deploy the French DA and the English DA	Knowledge mobilization

Activities throughout the process

Type: Project implementation follow-up meetings

Frequency: Every two weeks

Participants: Citizens partners, representatives from MSSS, representatives from DM & KMb affiliated to VITAM, CIUSSS-CN and Unité de Soutien SSA Québec

Tasks: Set up agenda, prepare and read working documents, hold meetings, write up meeting minutes

SC: Steering Committee
DA: Decision Aid
MSSS: *Ministère de la Santé et des Services sociaux* (Quebec Ministry of Health and Social services)
DM & KMb: Canada Research Chair in Shared Decision Making and Knowledge Mobilisation
VITAM: VITAM – *Centre de recherche en santé durable* (Sustainable health research in Québec)
CIUSSS-CN: *Centre Intégré Universitaire de Santé et de Services Sociaux de la Capitale Nationale* (Integrated University Health and Social Services Center of the Capitale-Nationale)
Unité de Soutien SSA Québec: *Unité de Soutien au système de santé apprenant Québec* (Learning healthcare system support unit Quebec)

Acknowledgments

Steering committee to develop the patient decision aid on lung cancer screening
Hervé Tchala Vignon Zomahoun, Claude Bernard Uwizeye, Anik Giguère,José Massougbodji, Nathalie Rheault, Wilhelm Dubuisson, Stéphane Groulx, Nicole Bouchard, Nicole Ezer, Simon Martel, EL Kebir Ghandour, Pamela Bou Malhab, Caroline Riou, Emmanuelle Bernard, Isabelle Théberge, Hélène Lizotte, Louise Légaré, Wilber Deck, France Légaré

Steering committee to develop the patient decision aid on colorectal cancer screening
Claude Bernard Uwizeye, Odilon Quentin Assan, Anik Giguère, Marie-Pierre Gagnon, Wilhelm Dubuisson, Mariejka Beauregard, Stéphane Groulx, Pamela Bou Malhab, Camille Poirier-Ouellet, Mélanie Robillard, Kim Landry-Truchon, Wilber Deck, Luc Ricard, Sophie Grignon, Alan Barkun, Charles Ménard, Jean Dubé, Mélanie Bélanger, France Légaré

Steering committee to develop the patient decision aid on cervical cancer screening
Claude Bernard Uwizeye, Odilon Quentin Assan, Samira Amil, Mariejka Beauregard, Sylvie Bouvet, Marie Cimon, Michel Cimon, Natalie Cormier, Wilber Deck, Wilhelm Dubuisson, Anik Giguère, Stéphane Groulx, Sabrina Guay-Belanger, Véronique Lebel, Caroline Riou, Mélanie Robillard, Guillaume Sillon, Annie Tremblay, France Légaré

English-language editor, for editing support
Louisa Blair

UNITÉ DE SOUTIEN SSA | QUÉBEC
Ensemble pour un système de santé qui apprend

VITAM
CENTRE DE RECHERCHE EN SANTÉ DURABLE

Chaire de recherche du Canada sur la décision partagée et la mobilisation des connaissances

UNIVERSITÉ LAVAL
